# 550 Considerations for Ophthalmologic Evaluation to Reduce Ocular Morbidity in Facial Burn Patients

**DOI:** 10.1093/jbcr/irac012.178

**Published:** 2022-03-23

**Authors:** Keith R Sweitzer, Jessica Forman, Derek Bell

**Affiliations:** University of Rochester, Rochester, New York; University of Rochester, Rochester, New York; University of Rochester, Rochester, New York

## Abstract

**Introduction:**

Advances in burn management have led to significant improvement in survival rates, even in patients with high total body surface area (TBSA) affected. Ocular morbidity in facial burn patients remains high, partially attributable to the life-threatening nature of these injuries. Previous studies have shown that early ophthalmologic intervention leads to better outcomes, however, specific risk factors for short and longer term ophthalmologic outcomes have not been elucidated. This study aimed to identify risk factors for short- and long-term ophthalmologic complications in facial burn patients to prioritize patients that require urgent ophthalmologic evaluation.

**Methods:**

Retrospective review of facial burn patients presenting to an American Burn Association-verified regional burn center between June 2007 and May 2016 was conducted. Demographics, presentation, time to ophthalmologic consultation, and short- and long-term complications were recorded. Odds ratio and multivariate analyses were performed to assess for significant risk factors.

**Results:**

A total of 1,126 facial burn patients were identified, of which 135 (12%) involved periorbital and orbital injury. Average TBSA burned was 9.68%, with an average facial surface area burned of 1.56%. The most common ocular injury was eyelid burn (65.9%). Ophthalmology was consulted for 118 (87.4%) patients. Short-term ophthalmologic complications were noted in 58 (43%) patients, most commonly chemosis (n = 34, 25.2%). Long-term complications were rare, occurring in only 7 (5.2%) patients.

Odds ratio analysis revealed that inhalation injury significantly increased the likelihood of both short- and long-term complications (OR 3.16 and OR 9.81, respectively). Active smoking increased the likelihood of long-term complications (OR 14.76). Ophthalmologic intervention, including need for consult, and use of lubricant, antibiotics, or steroids were each associated with increased risk of short-term complications.

On multivariate analysis, those with long-term complications tended to be older (p = 0.045). Those with corneal injury generally had worse outcomes, with higher likelihood of short- and long-term complications (p < 0.001, p = 0.057, respectively).

Blindness did not occur in any patient, and no long-term complications occurred in those who did not receive ophthalmologic consult. Neither TBSA nor facial SA burned was associated with the development of short- or long-term complications.

**Conclusions:**

Providers should obtain early ophthalmologic evaluation and frequent follow-up exams for facial burn patients presenting with advanced age, active smoking status, corneal injury, or inhalation injury to reduce development of long-term complications.

